# Carnitine palmitoyltransferase 2 deficiency, malignant hyperthermia and anesthesia

**DOI:** 10.1186/1471-2253-14-S1-A9

**Published:** 2014-08-18

**Authors:** Violeta Glauber, Haim Berkenstadt

**Affiliations:** 1 Department of Anesthesia and ICU, The Chaim Sheba Medical Center, Tel-Aviv University, Sackler School of Medicine, Tel-Hashomer, 52621, Israel

## Background

Carnitine palmitoyltransferase (CPT) deficiencies are a common autosomal recessive disorder resulting in a defect in mitochondrial fatty acid oxidation. The CPT system is made up of two separated proteins located in the outer CPT1 and inner CPT2 mitochondrial membranes. CPT1 catalyses the formation of acylcarnitine from carnitine and long chain fatty acyl-CoA. Acylcarnitine then crosses the inner mitochondrial membrane where it combines with CoA to form acyl-CoA, a process catalyzed by CPT2. Acyl-CoA is then available to undergo beta-oxidation. Deficiencies of both CPT1 and 2 have been described and leave patients unable to derive energy from fatty acid oxidation. Once immediate glucose and glycogen stores are exhausted, hypoglycemia may occur. This process conducts to rhabdomyolysis with muscle pains, hyperkalemia, metabolic acidosis and myoglobinuria. In severe severe cases acute renal failure, cardiac arrest and death ensure.

## Case report

A 35 years old woman was referred for a consultation to our malignant hyperthermia center before anesthesia for in vitro fertilization treatment (IVF). She never had an anesthesia. Her parents had 2 other healthy children. All family's members had had general anesthesia without problems. At 18 year old, after massive rhabdomyolysis during an interactive viral infection, the patient had a DNA analysis that revealed 1237Arg and Ser113Leu, mutations in CPT 2gene. The patient's was diagnosed as CPT2 deficiency. From these date she over went 4 crises of massive rhabdomyolisis, during which the CPK levels exceed 90.000 IU/l. The patient is in good physical status, renal functions are normal, liver enzymes are mildly elevated.

## Conclusions

1. Is there indication for muscle biopsy and in vitro contracture test (IVCT)? What is the risk and what is the benefit from IVCT?

2. Does this patient need a special genetic counseling before **pregnancy?**

3. IVF needs a great number of sedations - what is the sedation plan?

4. The pregnancy may aggravates her general status and the delivery may be a challenge for patient as well as for us - anesthesia plans.

